# Setting a shared development agenda: prioritizing the sustainable development goals in the Dominican Republic with fuzzy-LMAW

**DOI:** 10.1038/s41598-024-62790-w

**Published:** 2024-05-27

**Authors:** Luis A. Fernández-Portillo, Gülay Demir, Antonio Sianes, Francisco Santos-Carrillo

**Affiliations:** 1 Department of Business Management, Universidad Loyola Andalucía, Escritor Castilla Aguayo, 4, 14004 Córdoba, Spain; 2 Vocational School of Health Services, Sivas Cumhuriyet University, 58140 Sivas, Turkey; 3 Research Institute on Policies for Social Transformation, Universidad Loyola Andalucía, Escritor Castilla Aguayo, 4, 14004 Córdoba, Spain; 4 Department of International Studies, Universidad Loyola Andalucía, Escritor Castilla Aguayo, 4, 14004 Córdoba, Spain

**Keywords:** 2030 Agenda, Sustainable development goals (SDGs), Multi-criteria decision analysis (MCDA), Developing countries, Dominican Republic, Fuzzy LMAW, Sustainability, Socioeconomic scenarios

## Abstract

The sustainable development goals (SDGs) were established by the United Nations as an international call to eradicate poverty, safeguard the environment, and guarantee that everyone lives in peace and prosperity by 2030. The SDGs aim to balance growth and sustainability in three dimensions: social, economic and environmental. However, in the post-pandemic era, when resources for public development policies are scarce, nations face the problem of prioritizing which SDGs to pursue. A lack of agreement is one of the determinants of low performance levels of the SDGs, and multicriteria decision analysis tools can help in this task, which is especially relevant in developing countries that are falling behind in achieving the SDGs. To test the feasibility and appropriateness of one of these tools, the Fuzzy Logarithm Methodology of Additive Weights, we apply it to prioritize the SDGs in the Dominican Republic, to see if the priorities established are consistent. Seventeen experts were surveyed, and the main result was that Decent work and economic growth was the most important goal for the country. Our findings, consistent with the literature, show the path to similar applications in other developing countries to enhance performance levels in the achievement of the SDGs.

## Introduction

The 2030 Agenda for Sustainable Development was approved by the United Nations General Assembly in 2015 to promote sustainable development^1^. Considered a remarkable achievement of the international community, it was regarded an unprecedented global commitment^2^. In fact, it was negotiated and ratified by the immense majority of countries in the world and included in its mediation process other public bodies, civil society organizations and the private sector, who shared its global vision of the world^3^.

Shortly after the 2030 Agenda began to be implemented, it became clear that the needs of states were going to change from the design of the required institutional arrangements and procedures to the implementation of real actions^4^. This is even more evident now that we are halfway through the implementation period of the 2030 Agenda.

The most identifiable aspect of the 2030 Agenda is the 17 sustainable development goals (SDGs), which comprise 169 targets to be pursued from 2015 to 2030. They cover a wide range of sectors and challenges, from poverty and inequality to climate change, peace and global governance^5^. Such coverage reflects its scale and profound ambition but also explains the difficulties in its implementation and the consequent lack of critical pathways to success^6^.

If the main purpose of the Agenda, as declared, is to inform and orient public policies and private interventions, the identification of key SDGs able to mobilize both synergies between actors and funding is critical. In this context, prioritizing is paramount, given that the resources available are limited. For any country, the prioritization of goals and targets to be pursued will depend on the situation of each of the almost all-encompassing areas covered by the Agenda^7^. Policymakers find it difficult to translate the global goals stated in the Agenda into priority actions to be undertaken in their countries^8^. There is a risk that countries, especially those with a lower endowment of technical resources, prioritize SDGs and targets with arbitrary methods, following paths already trodden with limited success, choosing those targets easier to achieve^9^, or those in line with their development policies^10^. Thus, the international community is assuming that not all SDGs will be addressed in all countries, which calls for sectoral and geographical concentration of efforts^11^.

Several factors make the prioritization of the SDGs and targets especially difficult. First, the Agenda is multisectoral^12^. Second, its implementation should occur at different geographical and institutional levels^13^. Third, different types of stakeholders are involved^14^. Fourth, the SDGs are integrated and indivisible, i.e., they form a coherent and almost all-encompassing system of goals that countries should seek without renouncing any of them^1^. However, SDGs at the territorial scale are influenced by cultural, educational, and economic-social elements of the territory, which implies that many of the SDGs must be contextually ignored due to these constraints^15^. Fifth, the complexity generated by the multiple interactions among goals and targets is especially relevant^4,16,17^. Resolving current trade-offs between the SDGs to maximize synergies is crucial to guarantee their achievement^18^.

Additionally, COVID-19 has added more complexity to the implementation of the Agenda, firstly, because developed countries decided to redirect their efforts toward domestic needs. Secondly, because the limited endowment of public resources (financial and technical) in developing countries has forced the allocation of funds to fight the pandemic, often at the expense of other purposes^19^, so perhaps it should be adapted to address this type of global crisis^20^. Given their scarce endowment of resources and capabilities, developing countries will re-evaluate their priorities vis-a-vis the management of the pandemic and subsequent recovery strategies^21,22^. These facts add to a situation where international finance for development processes was already stagnant^23^.

In summary, the implementation of the Agenda in each country can be regarded as a decision-making problem in a complex context, which is especially difficult in developing countries with multiple and interacting criteria. This problem needs to be addressed with adequate tools able to address this complexity, such as multicriteria decision analysis (MCDA) techniques^24^. The use of fuzzy techniques is especially relevant because of their ability to address the vague, imprecise and subjective judgments needed to prioritize the components of the 2030 Agenda.

MCDA is based on the selection/ranking of the best alternative among the proposed alternatives using decision criteria that need to be prioritized. There are different methods for prioritizing criteria and/or alternatives, such as the Simple Multiattribute Ranking Technique (SMART)^25^, Analytic Hierarchy Process (AHP)^26^, Analytic Network Process (ANP)^27^, Best Worst Method (BWM)^28,29^, FUll COnsistency Method (FUCOM)^30,31^, goal programming^32,33^ and Logarithm Methodology of Additive Weights (LMAW)^34–36^.

Early enough^37^, it was understood the need for multicriteria methods to model the long-term planning of SDGs at the country level. Since then, several MCDA methods have been widely used concerning the 2030 Agenda for various purposes and in different ways.

Often, MCDA is used to measure the progress (for example, of a country) toward achieving the 2030 Agenda or the SDGs^38,39^. However, it is also possible that the connection between the 2030 Agenda and MCDA methods is more related to their role in decision-making. In this regard, and according to^8^, the Agenda and its SDGs can play three different roles when using an MCDA method: as criteria, for example, to prioritize projects or actors; as focus areas, providing the scope, context or research questions; and as alternatives. In this research, we refer to the latter.

Sousa et al.^24^ conducted a systematic review of the literature on the application of MCDA to the 2030 Agenda. They analyzed 143 papers, and apparently, only 5 dealt with all the SDGs, 13 with multiple SDGs, and the rest with only one SDG. The most frequently used method is AHP (73 papers), followed by Technique for Order of Preference by Similarity to Ideal Solution (TOPSIS) (31 papers).

From those 5 that address all the SDGs, we found that^40^ do not actually involve the 2030 Agenda or its SDGs in their analysis. On the other hand^41^, prioritized SDGs 1–15 in Turkey using neutrosophic Evaluation based on Distance from Average Solution (EDAS) (a fuzzy technique) and the opinions of 3 experts, with 4 criteria and 15 subcriteria. Oliveira et al.^16^ prioritized 32 targets (leaving out SDG 17, Partnerships of the goals) in Brazil, with 8 experts and 3 criteria; they used fuzzy AHP combined with other non-MCDA methods. Resce and Schiltz^42^ do not prioritize but measure the performance of the 17 SDGs in the European Union, using secondary data as inputs of the Hierarchical Stochastic Multicriteria Acceptability Analysis (HSMAA) method (they do not use criteria). Finally, ^43^ use secondary data with the opinions of 167 experts to prioritize all the targets of the 2030 Agenda in Switzerland. They combined AHP with other non-MCDA methods, but AHP is not used to prioritize SDGs but rather to decompose the decision into a hierarchy of more easily comprehensible subproblems.

Apart from the 5 papers quoted by^24^, ^8^ prioritized the SDGs (except SDGs 13, Climate action, and 17, Partnerships for the goals) in relation to climate action at the global level, with 31 experts, using a fuzzy version of TOPSIS combined with a non-MCDA method. Ranjbari et al.^21^ combined the BWM and a fuzzy inference system with a Delphi consultation with 19 experts to prioritize SDG targets in Iran with 4 criteria; after a filtering process, they prioritized targets from 10 SDGs. Toth et al.^14^ applied the ANP to re-evaluate the priorities calculated using secondary data with a non-MCDA method by Weitz et al.^4^ regarding 2 targets per SDG (34 targets in total) in Sweden, with 2 criteria. Deveci et al.^44^ prioritized SDGs that have more influence on the mining industry at the global level using the ordinal preference approach (a rough sets method) with inputs from 78 experts. This method does not use criteria to rank the elements, so in this case, the authors asked respondents to state their judgment on the level of importance of each SDG on a linguistic scale from very low to very high, without including any criteria to be considered in this judgment. Koasidis et al.^45^ prioritized the SDGs (except SDGs 13, Climate action; 16, Peace, justice and strong institutions, and 17, Partnerships for the goals) in relation to climate action in Kenya by applying a fuzzy version of TOPSIS to a group of 23 experts with 4 criteria.

From this review, we can see that most research papers use fuzzy methods to prioritize all or most of the SDGs, with a number of experts ranging from 3 to 167 and from 4 to 15 criteria, in a variety of geographical contexts, from developed countries (Sweden or Switzerland) to developing countries (Kenya, Brazil or Iran). Nevertheless, none of these studies prioritized the 17 SDGs in a developing country.

The participation of high-level stakeholders in an experiment of this kind, especially in a developing country, requires that the method used is not “cumbersome” nor time-consuming. We wonder if it is possible to use a single but robust method that is cost-efficient, accessible, versatile and user-friendly for bureaucracies with limited capacities and applicable to experts on the 2030 Agenda in a developing country to prioritize the implementation of the 17 SDGs.

Therefore, the main objective of this paper is to test the feasibility and appropriateness of the F-LMAW method and to illustrate its application with high-level experts in a developing country, the Dominican Republic (DR), to determine whether the priorities established by the experts are consistent.

The first contribution of this study is related to the method: our contribution shows the application of a relatively new method to an especially complex topic, such as the SDGs of the 2030 Agenda, characterized by its multi-dimensional nature, with conflicting objectives and various levels of implementation. The second contribution is showing the applicability of the method, as it opens the participation to stakeholders with different institutional backgrounds, something especially relevant in developing countries like those in Latin America, with agenda and time constraints. The third contribution is linked to the case study, DR: we acknowledge that this is a specific study, but given the selection reasons explained in “Materials and methods”, and although these results are not immediately generalizable to all Latin American countries, they likely reflect the situation in other middle-income and developing countries in a similar situation to the Dominican Republic, with high levels of national income with deep social inequalities. In any case, the application of the method to the case study is perfectly replicable in any country where there is a group of experts on the implementation of the 2030 Agenda available to participate. In fourth place, and regarding the results, now that we are halfway through the implementation period of the 2030 Agenda, the use of this type of sophisticated tool can be very relevant, especially in developing countries. It offers an agreed agenda to foster collective action.

The following section will address the methods and materials used in this research. “Results” section presents the results applying the method, which are discussed in “Discussion” section. The conclusions are presented in “Conclusions” section.

## Materials and methods

The LMAW method was introduced in the literature by Pamučar et al.^35^. LMAW was adapted by applying triangular fuzzy numbers by Božanić et al.^46^. The fuzzy version uses linguistic descriptors that are translated into fuzzy numbers (see Table 1).

**Table 1:** Fuzzy scale for criteria prioritization and evaluation of alternatives. *Source*:^68,69^.

Name of the fuzzy linguistic descriptor (criteria)	Abbreviation	Fuzzy number	Name of the Fuzzy linguistic descriptor (alternative)	Abbreviation	Fuzzy number
Absolutely low	AL	(1,1,1)	Very small	VS	(1,1,2)
Very low	VL	(1,1.5,2)	Small	S	(1,2,3)
Low	L	(1.5,2,2.5)	Middle	M	(2,3,4)
Medium	M	(2,2.5,3)	High	H	(3,4,5)
Equal	E	(2.5,3,3.5)	Very high	VH	(4,4,5)
Medium high	MH	(3,3.5,4)			
High	H	(3.5,4,4.5)			
Very high	VH	(4,4.5,5)			
Absolutely high	AH	(4.5,5,5)			

The main advantages of this method, which led us to choose it, are that it is more stable and reliable than methods based on similar principles (for example, TOPSIS); it does not cause problems of rank reversal (see below); the mathematical framework of the method remains the same regardless of the number of alternatives and criteria, being applicable to real-life situations with a high number of criteria; the same method is applied to weight criteria and rank alternatives, and it is sensitive to changes in the weights of criteria^35^.

For space reasons, a detailed description of the method is included in the Supplementary Information, but Table 2 summarizes the processing steps of the method, the equations used, and the result tables obtained at each step, including the sensitivity analysis.

**Table 2:** Summary of the steps of the LMAW method and the sensitivity analysis.

Step	Equation	Result
LMAW method		
Step 1. Creation of initial (expert) decision-making matrices	N.A.	Supplementary Table S1 (areas) Supplementary Table S2 (SDGs)*
Step 2. Creation of the initial (aggregated) decision-making matrices	Supplementary Eq. (1)	Supplementary Table S3 (areas) Supplementary Table S4 (SDGs)
Step 3. Normalization of the elements of the first decision-making matrices	Supplementary Eq. (2)	Supplementary Table S5 (areas) Supplementary Table S6 (SDGs)
Step 4. Determination of weight coefficients of criteria		
Step 4.1. Prioritization of criteria	N.A	Supplementary Table S7 (linguistic values) Supplementary Table S8 (numerical values)
Step 4.2. Identification of the absolute fuzzy anti-ideal point	N.A	${\widetilde{\gamma }}_{AIP}=$(0.5 0.5 0.5) has been adopted
Step 4.3. Identification of the fuzzy relationship vectors	Supplementary Eq. (3)	Supplementary Table S9
Step 4.4. Determination of vectors of the weight coefficients for each expert	Supplementary Eq. (4)	Supplementary Table S10
Step 4.5. Calculation of the aggregated fuzzy vector	Supplementary Eq. (5)	Supplementary Table S11
Step 4.6. Calculation of the final values of the weight coefficient	Supplementary Eq. (6)	Table 4
Step 5. Calculation of the weighted normalized matrix	Supplementary Eq. (7)	Supplementary Table S12 (areas) Supplementary Table S13 (SDGs)
Step 6. Calculation of the final index for the ranking of alternatives	Supplementary Eqs. (8)/(6)	Supplementary Table S14 (areas) Table 5 (SDGs)
Sensitivity analysis		
Step 1: Determination of the weight elasticity coefficient	Supplementary Eq. (9)	Supplementary Table S15
Step 2: Determination of the ∆x parameter	Supplementary Eq. (10)	Supplementary Table S15
Step 3. Calculation of new criteria weights	Supplementary Eqs. (11)/(12)	Figure 1, Supplementary Table S16
Step 4. Test of the “rank reversal problem”	N.A	Figure 2
Step 5. Comparison with other MCDA tools	Spearman rank correlation	Figure 3, Supplementary Table S17

*Given the extension of the individual decision-making matrices (17 matrices of 17 rows and 15 columns) with triangular fuzzy numbers, these cannot be included in the Supplementary Information, but are available at request.

Sensitivity analysis can be defined as the stability or behavior of the solution against small changes in preferences that occur during the solution process or minor changes in the values received for parameters^47^. There are various sensitivity analysis techniques, some of which are applied here^48^. In this case, the analysis tries to examine whether any change in the parameters of the model influences the ranking of the alternatives, to ensure the stability and robustness of the application^49^.

Firstly, we propose a weight sensitivity analysis, which is carried out by altering the weights of the most important criteria to determine how the suggested model affects the ranking performance^50^ (see Supplementary Information).

Secondly, adding new alternatives to the initial set or removing nonpreferred alternatives from the set are two strategies for testing the stability of MCDA models. In such cases, it is desirable that the MCDA technique not significantly affect the order of the alternatives. The “rank reversal problem” is the name given to this phenomenon and has been extensively discussed in the literature^51–53^. Creating dynamic matrices and analyzing the model’s reaction under the newly formed conditions is one method for evaluating the validity of the conclusions drawn from the decision-making model.

Thirdly, in many complex decision environments, sensitivity analysis is also performed by comparing the results of a model with those of other available and well-structured methods to examine the robustness and reliability of the ranking scores of the alternatives^54–56^. It explains how various MCDA methods can provide equivalent or dissimilar ranking scores by calculating the correlation coefficient between them.

Regarding the case study, the choice of the country is based on criteria of relevance, representativeness and accessibility to information. Firstly, the country has demonstrated its commitment to the Agenda as a planning horizon and state policy. It has aligned its policies with the SDGs and developed planning tools. On the other hand, it is a country that shares many of the structural problems of the region. Although it has relatively high development rates, it maintains high levels of inequality. In this sense, it has the potential to act as a disseminator of its experience. Finally, access to information sources has been a determining factor. The country’s history in the development of instruments and the authors’ previous experience allowed them to contact some of the national officials responsible for implementation, which represents an added value to this research.

The implementation of the 2030 Agenda in DR shows uneven progress with structural weaknesses. Two events in 2020 influenced the process: the pandemic and the change in government following the legislative elections in June.

Both aspects, critical to a successful implementation of the Agenda, call for multilevel and multistakeholder reflection to better understand the priorities of the DR in the years to come. The interview process described below attempts to show how this reflection could take place.

During July, August and September 2022, 9 public servants (PS) and 8 representatives of NGOs and International Cooperation (NGO/IC) institutions involved in the implementation of the 2030 Agenda in DR answered an online questionnaire about the priority of the 17 SDGs, which were classified into three areas: social (SDGs 1, 2, 3, 4, 5, 10), economic (SDGs 7, 8, 9, 11, 12) and environmental (SDGs 6, 13, 14, 15). SDGs 16 and 17 are considered cross-cutting, and although they were prioritized with the rest, they were not included in any of the three areas^57^. The research was approved by the Ethics Committee of the Universidad Loyola Andalucía, following its guidelines and regulations. Informed consent was obtained from all participants.

The evaluation criteria used to evaluate the alternatives, the SDGs and the three areas were relevance (C1), urgency (C2), convergence (C3), alignment (C4) and independence (C5). Their meanings are explained in Table 3. These criteria were discussed and agreed upon with the participants, who considered them relevant for the analysis. The relevance criterion must be understood as the importance that the experts think the area or the SDG should have for the country (therefore, this criterion has a normative role). The same occurs with urgency, whereas the last three criteria refer to the actual situation in the country (descriptive role). It was made clear to the experts that when the criteria convergence and alignment refer to policies/plans/partners, it was required that they are being implemented or working, not merely stated on paper.

**Table 3:** Explanation of decision criteria.

Criterion	Meaning
Relevance	Importance that the achievement of the goal has for the Dominican Republic
Urgency	Urgent need for the Dominican Republic to address the goal
Convergence	Measures whether the goal is consistent with policies/plans/partners in the Dominican Republic that can facilitate its achievement
Alignment	Measures the existence of policies/plans/partners in the Central American region that can support the achievement of the goal in the Dominican Republic
Independence	Possibility that the Dominican Republic can achieve the goal autonomously and without the need for cooperation or assistance from others

The respondents were heads of departments or similar positions (9), representatives of their institution in the country (2), and technical specialists on the Agenda or some of its main areas (6). Given their positions, it can be said that the respondents’ opinions about the Agenda are likely to be well founded on their knowledge about it.

## Results

The F-LMAW method is applied in three stages: weighting of the evaluation criteria, weighting of social, economic and environmental areas, and SDGs ranking. Then, a sensitivity analysis is performed.

### Weighting of evaluation criteria

The first matrix is generated based on the linguistic evaluation provided by the experts to the criteria (see Supplementary Tables S7 and S8). Then, the relationship between each element of the priority vectors of the five criteria and the absolute anti-ideal point (AIP) was defined. For this purpose, ${\widetilde{\gamma }}_{AIP}=$ (0.5 0.5 0.5) was adopted (see Supplementary Table S9). The values of the weight coefficient vectors of the criteria are given in Supplementary Table S10. The final weighted coefficients of the criteria and their normalized values are given in Table 4.

**Table 4:** Final weights and normalized values of the criteria.

Criteria	Final weight coefficients	Normalized weight coefficients
C1	0.21465	0.21465/1.00226 = 0.2142
C2	0.20634	0.20634/1.00226 = 0.2059
C3	0.20360	0.20360/1.00226 = 0.2031
C4	0.18687	0.18687/1.00226 = 0.1865
C5	0.19079	0.19079/1.00226 = 0.1904
Total	1.00226	1.0000

### Weighting of social, economic and environmental areas

The first decision matrix of the three areas is presented in Supplementary Table S3, based on the linguistic evaluations for the three dimensions (Supplementary Table S1). The normalized decision matrix is presented in Supplementary Table S5. The weighted normalized matrix is shown in Supplementary Table S12. The final index values Qi of the dimensions were calculated by applying Supplementary Eq. (8). In this case, the most important area is social (Qi = 4.2402), followed by economic (Qi = 4.2390) and environmental (Qi = 4.1602) (Supplementary Table S14).

### Ranking the SDGs

The first decision matrix is presented in Supplementary Table S4, based on the linguistic answers of the experts (Supplementary Table S2). The normalized decision matrix is displayed in Supplementary Table S6, and the weighted normalized matrix is displayed in Supplementary Table S13. The final index values of the alternatives were also determined by applying Supplementary Eq. (8). These values were considered to determine the SDGs’ final ranking, shown in Table 5.

**Table 5:** Final index for the ranking of the SDGs (fuzzy and defuzzified).

	${\widetilde{{\varvec{Q}}}}_{{\varvec{i}}}$			${{\varvec{Q}}}_{{\varvec{i}}}$	Rank
SDG1	3.0335	3.3395	3.6856	3.3462	3
SDG2	3.0095	3.3388	3.6934	3.3430	4
SDG3	3.0061	3.3219	3.6782	3.3287	10
SDG4	3.0286	3.3319	3.6801	3.3394	5
SDG5	3.0067	3.3294	3.6849	3.3348	7
SDG10	2.9855	3.3058	3.6694	3.3130	13
SDG7	2.9497	3.2981	3.6738	3.3027	14
SDG8	3.0352	3.3476	3.6942	3.3533	1
SDG9	2.9560	3.3131	3.6861	3.3157	12
SDG11	2.9329	3.2951	3.6777	3.2985	15
SDG12	2.9485	3.2834	3.6594	3.2902	16
SDG6	3.0283	3.3248	3.6727	3.3334	8
SDG13	3.0032	3.3246	3.6821	3.3306	9
SDG14	2.9389	3.2791	3.6581	3.2856	17
SDG15	2.9738	3.3177	3.6852	3.3217	11
SDG16	3.0457	3.3452	3.6882	3.3525	2
SDG17	2.9979	3.3331	3.6925	3.3371	6

An independent sample Student’s t-test was applied to determine whether there was a significant difference between the scores of PS and NGO/IC experts. The analysis (sig. (2-tailed) = 0.982 > 0.05) revealed that there was no significant difference.

### Sensitivity analysis

A three-step sensitivity analysis was performed to test the stability and sensitivity of the proposed method. In the first stage, the effect of changing the criteria weights on the ranking results was examined to ensure the stability and robustness of the application. Next, the effect of the rank reversal matrix was tested. Finally, four MCDA techniques were applied and compared with the results of the proposed method.

**Figure 1:**
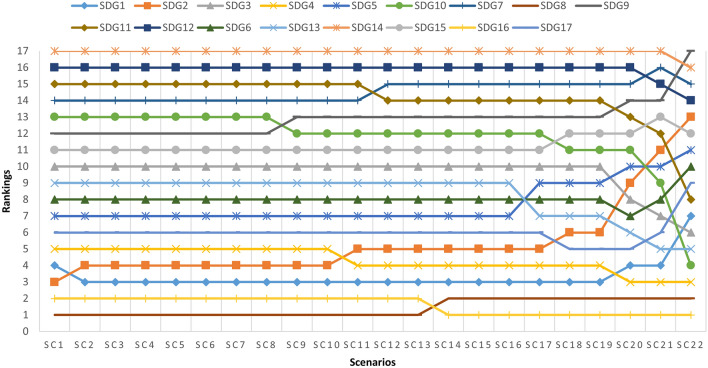
Changes in SDG rankings. Assigning different weights to criteria causes a change in the ranking of all SDGs.

In relation to the first analysis, the ${\alpha }_{c}$ value of the weighting coefficient of the most important criterion (here, C1) is taken as 1. It is estimated using Supplementary Eq. (9) and shown in Supplementary Table S15. Then, the weight change limits (∆x) for the C1 criterion are calculated. They lie between − 0.2142 and 0.7858. For the first scenario, the minimum value of ∆x = − 0.2142 is taken, and in each subsequent scenario, the minimum value is increased by 0.05 until the maximum value of ∆x = 0.7858 is reached, and Supplementary Eqs. (11) and (12) are used, as shown in Supplementary Table S16. New weights were calculated with 22 sets of scenarios. The rankings obtained by recalculating the priorities of the SDGs with the 22 weight sets are given in Fig. 1.

Figure 1 shows that assigning different weights to criteria across 22 scenarios causes a change in the ranking of all SDGs. Thus, it has been verified that the model is sensitive to changes in weight coefficients.

**Figure 2:**
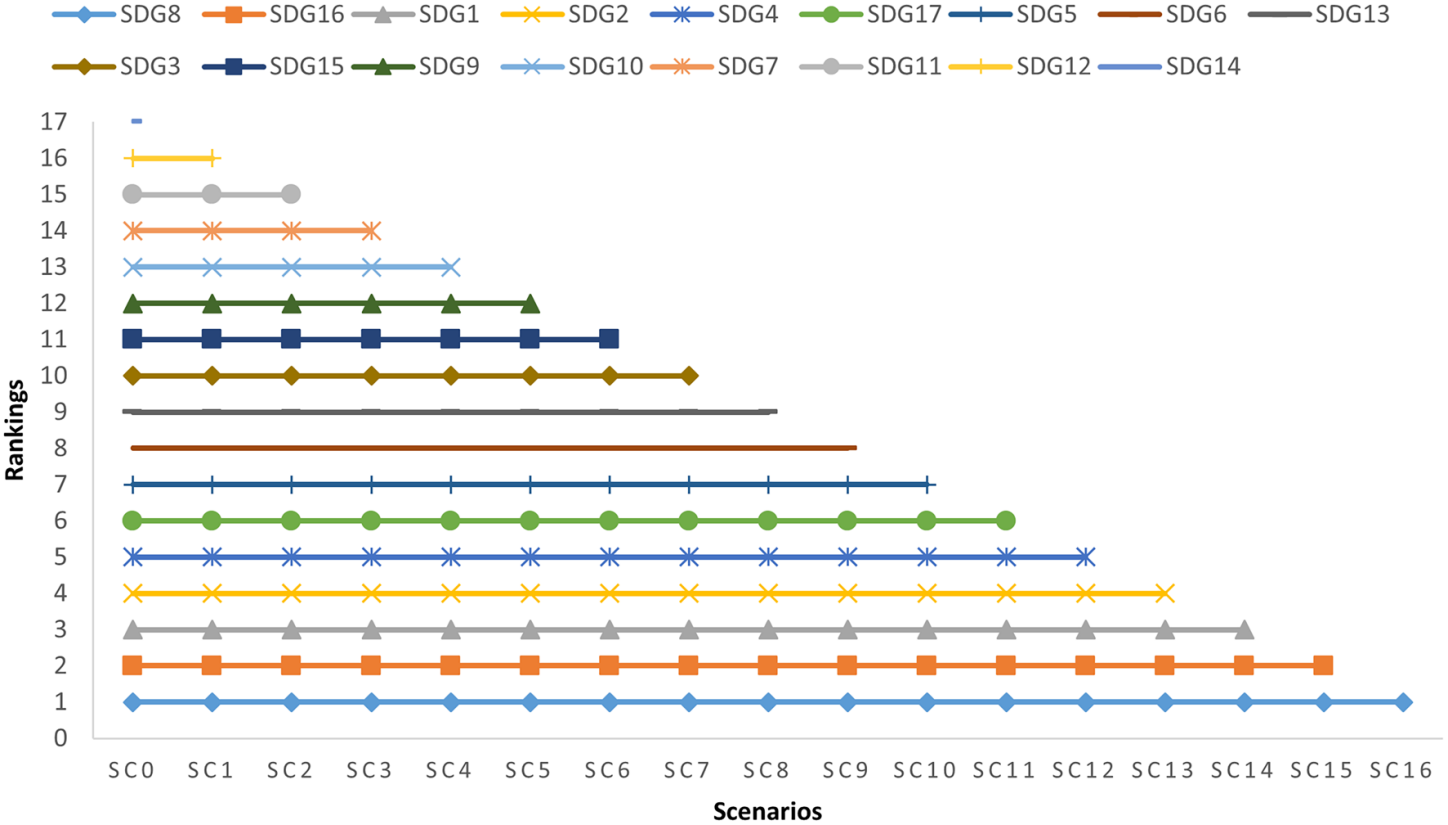
Results of the rank reversal matrix. The remaining SDGs maintain the same order of prevalence in the resulting rankings once the last SDG from the group in each subsequent scenario is eliminated.

Second, a test was run that considered the model’s resilience to the rank reversal problem. Sixteen scenarios were constructed for the test in which the modification of the decision matrix components was simulated by subtracting one SDG each time to create a new scenario. The SDGs are then rated using the F-LMAW approach, with Sc0 being the original ranking. SDG14, Life below water, ranked last in the original scenario, is eliminated in Sc1. The remaining 16 SDGs are then ranked again. A total of 16 situations (Sc1–Sc16) are produced by removing the last SDG from the group in each subsequent scenario. The results show that the remaining SDGs maintain the same order of prevalence in the resulting rankings, proving that the F-LMAW model delivers reliable rankings and is strongly resistant to the rank reversal problem (see Fig. 2).

**Figure 3:**
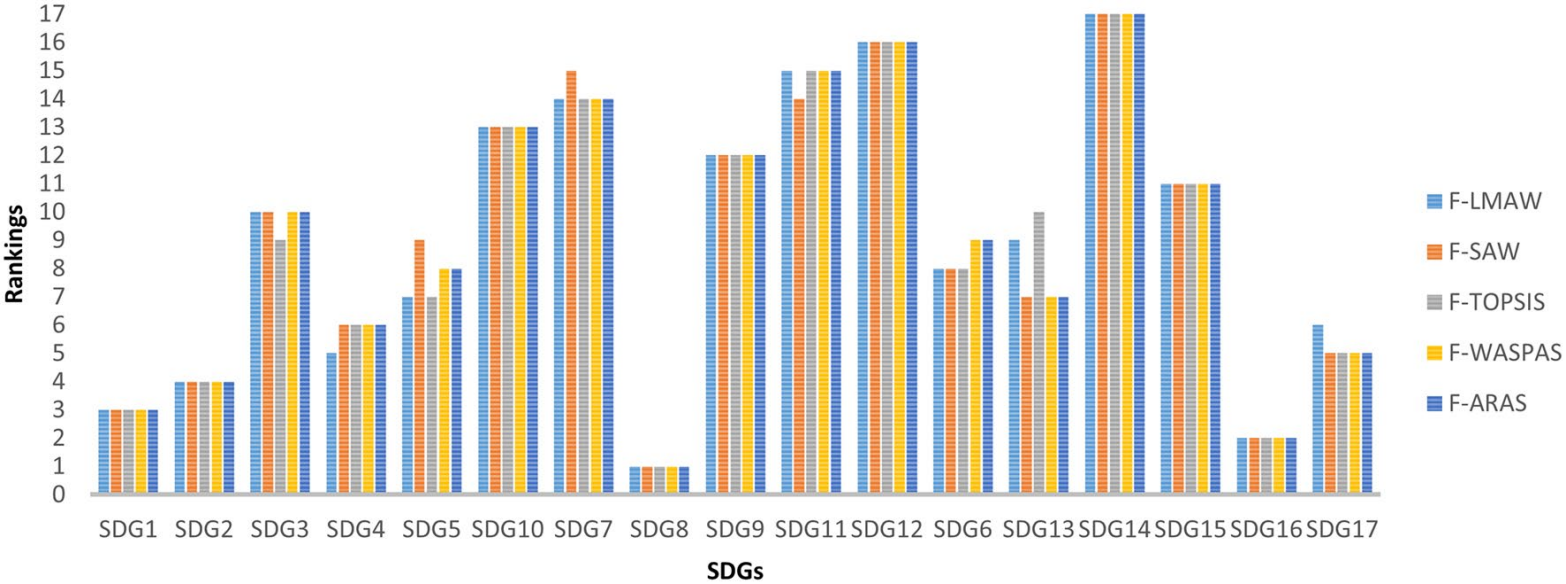
Rankings of SDGs by different MCDA methods. The rankings obtained with different MCDA methods are similar.

A comparison study using other MCDA techniques was performed to determine the stability of the ranking. Widely used approaches, such as F-SAW^58^, F-TOPSIS^59^, F-WASPAS^60^, and F-ARAS^61^, were used. The results of these methods are shown in Fig. 3.

Spearman Rank Correlation was used to compare the ranking performances obtained by different methods. With a correlation value higher than 0.98 (see Supplementary Table S17) between the other four MCDA techniques and the F-LMAW approach, the ranking obtained was verified and reliable.

## Discussion

The most important criterion was *relevance* (C1), followed by *urgency* (C2). They are the only criteria that reflect the subjective priorities of the experts. The lowest priority criterion is *alignment*, which measures the link between an SDG or area and initiatives from the Central American region. Thus, this result confirms a certain disaffection of Dominican bureaucracies regarding Central American regional institutions. It is a question of identity, distrust and a still insufficient shared history. It is not reciprocal because regional institutions distribute resources equitably, and practically all Central American Integration System (SICA) institutions have Dominican public servants^62^. Notably, the DR was the last to join the SICA and is the only country physically out of the isthmus. Criterion C5, *independence*, was fourth in priority. Some experts noted that the main factor here was the low endowment of technical resources rather than a lack of financial means.

Regarding the areas, *Environmental* receives the lowest priority. As is the case for most small developing countries, they feel that they are more victims of the pollution of others than polluters and, therefore, their investment in this area may yield scarce results^8,63^.

The *Social* area was found to be more important for the country, although many of the SDGs included in it also have an economic profile. Nevertheless, the most prioritized goal is SDG8, Decent work and economic growth, expressing the concern of the respondents about the need to generate income for the country to address the different challenges it is facing, exacerbated by the pandemic. We can translate these results as a will to generate economic growth but with a clear social outlook consistent with the most recent declarations of public bodies in the country.

Thus, it is slightly surprising that the *Economic* area did not rank first, especially because 7 of the 17 experts were public servants of two ministries with an economic scope. Although experts from more socially involved institutions, such as NGOs, showed an evident and understandable bias toward the *Social* area, the answers of these 7 experts also reflected a concern for social issues. The fact that 3 of the 6 SDGs in the *Social* area have a clear link to the economy (SDG1, No poverty; SDG2, Zero hunger; and SDG10, Reduced inequalities) may also explain this circumstance^11,64^.

Regarding the ranking of the SDGs, most experts declared that prioritizing SDGs and areas was a necessary task but that they understood the 2030 Agenda as a whole, with many interactions among goals and targets that they tried to keep in mind during the exercise. For example, if a given SDG may positively impact other SDGs, it should be more important. This fact showed, perhaps, that there was a criterion not considered by the authors: the ability of the SDGs to impact other SDGs. This emerged criterion is consistent with the literature on the field that calls for finding synergies between SDGs^9,17,18,65^ and points to a deep knowledge of the 2030 Agenda by the respondents.

As mentioned, SDG8, Decent work and economic growth, has the highest priority. Some experts pointed to the country’s high percentage of informal economy and deplorable labor conditions. Additionally, they expressed their concern about the economic stress caused by immigration, which could endanger the economic development of the country. The pandemic has also slowed the achievement of other SDGs, as the last Voluntary National Review on the achievement of the SDGs states^66^. Their support for this goal is due to the will to improve economic development quantitatively and qualitatively, and to the potential of this goal to impact other goals^11^. This explains why the public call to the private sector to support the SDGs is a national priority, in line with the potential of innovative businesses as a development lever^67^.

On the other hand, among the 6 lowest ranked SDGs, 4 are economic (SDG9, Industry, innovation and infrastructure; SDG7, Affordable and clean energy; SDG11, Sustainable cities and communities, and SDG12, Responsible consumption and production). According to the analysis by Forestier and Kim^10^, these goals are prioritized by 0% of the upper-middle-income countries (UMIs) (DR is classified there by the World Bank).

SDG16, Peace, justice and strong institutions, is ranked second. This finding is consistent with the analysis by Forestier and Kim^10^, as 20% of UMIs include that SDG in their priorities. Most of the experts commented on the need to strengthen public institutions, which were weakened by corruption. Otherwise, the achievement of other SDGs will be compromised. This shows the experts’ awareness about the crucial role of the public sector in achieving the SDGs, as they are expected to act as orchestrators^14^.

SDG1, No poverty, and SDG2, Zero hunger, rank third and fourth, respectively. Some experts expressed that poverty exists in the country mainly not because of a lack of wealth but due to its unequal distribution. Therefore, SDGs 1 and 2 should have lower priority than SDG10 (Reduced inequalities). However, the results show that SDG10 is ranked 13th. A possible explanation, suggested by some experts, is that they feel that it is easier to achieve SDGs 1 and 2 than to achieve SDG10, given the corruption already mentioned and the reluctance of the elites to yield power. These results are also consistent with those of^10^.

The low ranking of SDG14, Life below water, may be because the experts may feel that the country has scarce capacity to improve this goal^8^ in a situation where DR is already making a comparatively greater effort than the surrounding countries.

## Conclusions

The application of F-LMAW to the DR case study provides useful guidance not only on how to set a shared development agenda in the country but also on relevant insights in different fields.

Regarding the main objective, this study highlights that F-LMAW is a helpful tool that can be used to model the implementation of the SDGs as an MCDA problem and can prioritize and rank the SDGs, which is crucial to the successful implementation of the 2030 Agenda^21,22,24^. Our case study shows how the F-LMAW model delivers reliable findings in a dynamic setting and that it is strongly resistant to the rank reversal problem. It also shows the highest consistency with the other four MCDA techniques, which proves that the ranking obtained with F-LMAW is both verified and reliable.

This is very good news for the field of policy science. The priority and rank of the SDGs and the criteria were determined using the same method in a fuzzy environment in which linguistic descriptors were easier for the respondents to understand, as they somehow reflected the way they thought of priorities. It is worth noting that the experts participating in the experiment felt comfortable answering the questions and understood the need to consider the interactions between goals. The process of collecting their opinions was also found to be useful for them as a learning exercise because they had to reflect on the current situation of different factors that hinder or support the implementation of the SDGs (related to the last three criteria), as well as on the highest priority SDGs according to the needs of DR (related to the first two criteria).

The implementation of the study shows two limitations. First, regarding criteria, there was a criterion not considered by the authors: the ability of the SDGs to impact other SDGs. It was partially covered due to the respondents’ deep knowledge of the 2030 Agenda. However, in future applications, it should be included as a criterion. Second, regarding participants, representatives from the business sectors were absent in the sample. This absence was due to attempts to contact several business sector representatives who did not respond to our inquiries. Given the exceptional circumstances during the pandemic, it was challenging to address this issue. In future research, it would be beneficial to include such stakeholders in the analysis.

Despite these limitations, and although these results are “country-specific,” they are likely to reflect the situation in DR and also in other upper-middle-income and developing countries in a similar situation: high levels of national income with deep social inequalities. Nevertheless, the method is perfectly applicable to any country where a group of experts on the implementation of the 2030 Agenda are available to participate, as it has proven both feasible and advisable.

### Supplementary Information

Supplementary Information.

## Data Availability

Main data are available as Supplementary Information. The rest of the data are available upon request.
