# Supplementary material for: Setting a shared development agenda: prioritizing the sustainable development goals in the Dominican Republic with fuzzy-LMAW
Source: Sci Rep. 2024 May 27;14:12146. doi: 10.1038/s41598-024-62790-w (PMC11130273; doi:10.1038/s41598-024-62790-w)
Supplement: Supplementary file 1 — Supplementary Information. [file 41598_2024_62790_MOESM1_ESM.pdf]

The LMAW Method

The LMAW method was introduced to the literature by [1]. LMAW was adapted by applying triangular fuzzy numbers by [2]. The main advantages of this method that led us to choose it are that it is more stable and reliable than methods based on similar principles (for example, TOPSIS); it does not cause problems of rank reversal (see below); the mathematical framework of the method remains the same regardless of the number of alternatives and criteria, being applicable to real-life situations with a high number of criteria; the same method is applied to weight criteria and rank alternatives, and it is sensitive to changes in the weights of criteria [1].

The processing steps of the method are as follows, considering that in this case, the prioritization was carried out at two levels. On the one hand, the three areas were prioritized, followed by the SDGs.

Step 1. Creation of initial (expert) decision-making matrices $\left(\tilde{X}^{e}\right)$

Each expert e from the group of k experts defines an initial decision-making matrix in which evaluates m alternatives in relation to n criteria (areas and SDGs). Accordingly, the matrix $\tilde{X}^{e} = \left[\tilde{\vartheta}_{ij}^{e}\right]_{mxn}$is obtained for each expert, where $\tilde{\vartheta}_{ij}^{e}$ presents the fuzzy value by which expert e evaluates the criteria j and the i alternative.

Step 2. Creation of the initial (aggregated) decision-making matrix $\left(\tilde{X}\right)$

The summation of the initial (experts) matrices into an aggregated matrix is done by applying the Bonferroni aggregator according to Supplementary Equation (1)

$$\tilde{\vartheta}_{ij} = \left(\frac{1}{k(k-1)} \sum_{\substack{i,j=1 \\ i\neq j}}^{k} \tilde{\vartheta}_{i}^{(e)p} \tilde{\vartheta}_{j}^{(e)q} \right) =$$

$$\left\{ \left(\frac{1}{k(k-1)} \sum_{\substack{i,j=1 \\ i\neq j}}^{k} \vartheta_{i}^{(l_e)p} \vartheta_{j}^{(l_e)q} \right)^{\frac{1}{p+q}}, \left(\frac{1}{k(k-1)} \sum_{\substack{i,j=1 \\ i\neq j}}^{k} \vartheta_{i}^{(m_e)p} \vartheta_{j}^{(m_e)q} \right)^{\frac{1}{p+q}}, \left(\frac{1}{k(k-1)} \sum_{\substack{i,j=1 \\ i\neq j}}^{k} \vartheta_{i}^{(r_e)p} \vartheta_{j}^{(r_e)q} \right)^{\frac{1}{p+q}} \right\} \quad (1)$$

Here, $\tilde{\vartheta}_{ij}$ presents the average values obtained by applying the Bonferroni aggregator; $p, q \geq 0$ presents the stabilization parameters of the Bonferroni aggregator. Before the aggregation, the quantification of the linguistic values is carried out.

For example, the numerical value of $\tilde{\vartheta}_{11}$, the first element of the areas' first decision matrix, is the Bonferonni mean of the 17 experts' preferences:

$\tilde{\vartheta}_{11} = (\mathrm{VH, VH, VH, VH, H, VH, VH, VH, VH, VH, VH, VH, VH, VH, VH, H, VH}) = (4,4,5\ 4,4,5, \dots, 3,4,5, 4,4,5)$

Equation (1) is calculated as follows.

$$\tilde{\vartheta}_{11} = \begin{cases} \sqrt{\dfrac{1}{17 * (17-1)} * \{(4 * (4 + 4 + \cdots + 3 + 4) + \cdots + 4 * (4 + 4 + \cdots + 4 + 3)\},} \\ \sqrt{\dfrac{1}{17 * (17-1)} * \{(4 * (4 + 4 + \cdots + 4 + 4) + \cdots + 4 * (4 + 4 + \cdots + 4 + 4)\},} \\ \sqrt{\dfrac{1}{17 * (17-1)} * \{(5 * (5 + 5 + \cdots + 5 + 5) + \cdots + 5 * (5 + 5 + \cdots + 5 + 5)\}} \end{cases}$$

$\tilde{\vartheta}_{11} = (7.4449\ 7.9118\ 12.3621)$

Step 3. Normalization of the elements of the first decision-making matrix

When Supplementary Equation (2) is applied, the normalized matrix $\tilde{\Upsilon} = \left[\tilde{\vartheta}'_{ij}\right]_{mxn}$ is obtained.

$$\tilde{\vartheta}'_{ij} = \begin{cases} 1 + \dfrac{\tilde{\vartheta}_{ij}}{\tilde{\vartheta}_j^{(+)}} = \left(1 + \dfrac{\vartheta_{ij}^{(l)}}{\tilde{\vartheta}_j^{(+)}}, 1 + \dfrac{\vartheta_{ij}^{(m)}}{\tilde{\vartheta}_j^{(+)}}, 1 + \dfrac{\vartheta_{ij}^{(r)}}{\tilde{\vartheta}_j^{(+)}}\right) if\ j \in \text{benefit} \\ 1 + \dfrac{\tilde{\vartheta}_j^{(-)}}{\tilde{\vartheta}_{ij}} = \left(1 + \dfrac{\tilde{\vartheta}_j^{(-)}}{\vartheta_{ij}^{(r)}}, 1 + \dfrac{\tilde{\vartheta}_j^{(-)}}{\vartheta_{ij}^{(m)}}, 1 + \dfrac{\tilde{\vartheta}_j^{(-)}}{\vartheta_{ij}^{(l)}}\right) if\ j \in \text{cost} \end{cases} \quad (2)$$

Here, $\tilde{\vartheta}'_{ij}$ represents the normalized values of the first decision-making matrix, $\tilde{\vartheta}_j^{(+)} = max\left(\vartheta_j^{(r)}\right)$ and $\tilde{\vartheta}_j^{(-)} = min\left(\vartheta_j^{(l)}\right)$.

The following formula is used to determine the values of $\tilde{\vartheta}'_{11}$ in the normalized matrix of the areas.

$$\tilde{\vartheta}'_{11} = \left(1 + \frac{7.4449}{12.3621}, 1 + \frac{7.9118}{12.3621}, 1 + \frac{12.3621}{12.3621}\right) = (1.6022\ 1.6400\ 2.000)$$

Step 4. Determination of weight coefficients of criteria

In this step, the group of experts prioritize the decision criteria according to the linguistic evaluation scale given in Table 1.

Step 4.1. Prioritization of criteria. Based on the values on the fuzzy linguistic scale, experts prioritize the criteria $C = \{C_1, C_2, \dots, C_n\}$. In this way, the priority vectors $\tilde{P}^e = \left(\tilde{\gamma}_{C_1}^e, \tilde{\gamma}_{C_2}^e, \dots, \tilde{\gamma}_{C_n}^e\right)$ are defined separately for each expert, where $\tilde{\gamma}_{C_n}^e$ presents the value from the fuzzy linguistic scale that expert e assigns to the criteria.

Step 4.2. Identification of the absolute fuzzy anti-ideal point ($\tilde{\gamma}_{AIP}$). This value is defined by a decision maker, and it is a fuzzy number that is smaller than the smallest value in the entire set of priority vectors. In this case $\tilde{\gamma}_{AIP}$= (0.5 0.5 0.5) has been adopted.

Step 4.3. Identification of the fuzzy relationship vector $\left(\tilde{R}^e\right)$. By applying Supplementary Equation (3), the relationship between the elements of the priority vector and the absolute anti-ideal point $\tilde{\gamma}_{AIP}$ is determined for each expert.

$$\tilde{\eta}_{C_n}^e = \left(\frac{\tilde{\gamma}_{C_n}^e}{\tilde{\gamma}_{AIP}}\right) = \left(\frac{\gamma_{C_n}^{(l)e}}{\gamma_{AIP}^{(r)}}, \frac{\gamma_{C_n}^{(m)e}}{\gamma_{AIP}^{(m)}}, \frac{\gamma_{C_n}^{(r)e}}{\gamma_{AIP}^{(l)}}\right) \tag{3}$$

For example, the relationship between the priority vector elements defined by e1 and the absolute antiideal point is calculated as follows.

$$\tilde{\eta}_{C1}^{e1} = \left(\frac{4.5}{0.5}, \frac{5}{0.5}, \frac{5}{0.5}\right) = (9,10,10), \tilde{\eta}_{C2}^{e1} = \left(\frac{4}{0.5}, \frac{4.5}{0.5}, \frac{5}{0.5}\right) = (8,9,10), \tilde{\eta}_{C3}^{e1} = \left(\frac{2.5}{0.5}, \frac{3}{0.5}, \frac{3.5}{0.5}\right) = (5,6,7),$$

$$\tilde{\eta}_{C4}^{e1} = \left(\frac{3.5}{0.5}, \frac{4}{0.5}, \frac{4.5}{0.5}\right) = (7,8,9), \tilde{\eta}_{C5}^{e1} = \left(\frac{4}{0.5}, \frac{4.5}{0.5}, \frac{5}{0.5}\right) = (8,9,10)$$

For the other experts, calculations are made in a similar way.

Step 4.4. Determination of vectors of the weight coefficients of $w_j^e = (\tilde{w}_1^e, \tilde{w}_2^e, \dots, \tilde{w}_n^e)^T$ for each expert individually. The fuzzy values of the weight coefficients of the criteria for expert e are obtained by applying Supplementary Equation (4).

$$\tilde{w}_j^e = \left(\frac{ln(\tilde{\eta}_{C_n}^e)}{ln(\prod_{j=1}^n \tilde{\eta}_{C_n}^e)}\right) = \left(\frac{ln\left(\eta_{C_n}^{(l)e}\right)}{ln\left(\prod_{j=1}^n \eta_{C_n}^{(r)e}\right)}, \frac{ln\left(\eta_{C_n}^{(m)e}\right)}{ln\left(\prod_{j=1}^n \eta_{C_n}^{(m)e}\right)}, \frac{ln\left(\eta_{C_n}^{(r)e}\right)}{ln\left(\prod_{j=1}^n \eta_{C_n}^{(l)e}\right)}\right) \tag{4}$$

Here, $\tilde{\eta}_{C_n}^e$ represents the $\tilde{R}^e$ elements of the relationship vector, $\eta_{C_n}^{(l)e}$ represents the left distribution of the fuzzy priority vector, $\eta_{C_n}^{(r)e}$ is the right distribution of the fuzzy priority vector, and $\eta_{C_n}^{(m)e}$ is the value where the membership function of the fuzzy priority vector is equal to one.

Here is an example of calculating the weight coefficient vector for e1 according to the C1 criterion:

$$\widetilde{w}_1^1 = \left(\frac{\ln(9)}{\ln(10*10*7*9*10)}, \frac{\ln(10)}{\ln(10*9*6*8*9)}, \frac{\ln(10)}{\ln(9*8*5*7*8)}\right) = (0.19883\ 0.21788\ 0.23232)$$

Other values of the weight coefficients of the criteria are similarly calculated.

Step 4.5. Calculation of the aggregated fuzzy vectors $w_j = (\widetilde{w}_1, \widetilde{w}_2, \dots, \widetilde{w}_n)^T$ of weight coefficients. The aggregated fuzzy vectors of the weight coefficients are obtained by applying the Bonferroni aggregator according to Supplementary Equation (5).

$$\widetilde{w}_j = \left(\frac{1}{k(k-1)}\sum_{\substack{i,j=1\\ i\neq j}}^{k} \widetilde{w}_i^{e(p)} \widetilde{w}_j^{e(q)}\right)^{\frac{1}{p+q}} =$$

$$\left\{\left(\frac{1}{k(k-1)}\sum_{\substack{i,j=1\\ i\neq j}}^{k} w_i^{(l_e)p} w_j^{(l_e)q}\right)^{\frac{1}{p+q}}, \left(\frac{1}{k(k-1)}\sum_{\substack{i,j=1\\ i\neq j}}^{k} w_i^{(m_e)p} w_j^{(m_e)q}\right)^{\frac{1}{p+q}}, \left(\frac{1}{k(k-1)}\sum_{\substack{i,j=1\\ i\neq j}}^{k} w_i^{(r_e)p} w_j^{(r_e)q}\right)^{\frac{1}{p+q}}\right\} \quad (5)$$

Here, $p, q \geq 0$ represent the stabilization parameters of the Bonferroni aggregator, $w_j^{(l_e)}$ represents the left distribution of the fuzzy weight coefficient $\widetilde{w}_j^e, w_j^{(r_e)}$ represents the right distribution of the fuzzy weight coefficient $\widetilde{w}_j^e$, and $w_j^{(m_e)}$ represents the value where the membership function of the fuzzy weight coefficient $\widetilde{w}_j^e$ is equal to one.

Here is an example of the aggregated weight coefficient vector for criterion C1.

$$\widetilde{w}_1 = \begin{cases} \sqrt{\frac{1}{17*(17-1)} * \{(0.19833*(0.20045+0.19441+\dots+0.19943+0.21000)+\dots+0.21000*(0.19883+\dots+0.19943)\}}, \\ \sqrt{\frac{1}{17*(17-1)} * \{(0.21788*(0.21990+0.21007+\dots+0.23284+0.23071)+\dots+0.23071*(0.21788+\dots+0.23284)\}}, \\ \sqrt{\frac{1}{17*(17-1)} * \{(0.23231*(0.23481+0.22213+\dots+0.27825+0.25077)+\dots+0.25077*(0.23232+\dots+0.27825)\}} \end{cases}$$

$\widetilde{w}_1 = (0.19407\ 0.21470\ 0.23501\ )$

Step 4.6. Calculation of the final values of the weight coefficient $w_j = (w_1, w_2, \dots, w_n)^T$. The final values of the weight coefficients of the criteria are obtained by defuzzification according to Supplementary Equation (6).

$$w_j = \frac{l + 4m + r}{6} \tag{6}$$

Here is an example of calculating the weight coefficient for criterion C1:

$$w_1 = \frac{0.19407 + 4 * 0.21470 + 0.23501}{6} = 0.21465$$

Step 5. Calculation of the weighted normalized matrix (N)

The elements of the $N = [\xi_{ij}]_{mxn}$ weighted normalized matrix are obtained by applying Supplementary Equation (7).

$$\xi_{ij} = \frac{2 * \tilde{\varphi}_{ij}^{w_j}}{(2 - \tilde{\varphi}_{ij})^{w_j} + \tilde{\varphi}_{ij}^{w_j}}$$

$$= \left(\frac{2 * \varphi_{ij}^{(l)^{w_j}}}{\left(2 - \varphi_j^{(r)}\right)^{w_j} + \varphi^{(r)^{w_j}}}, \frac{2 * \varphi_{ij}^{(m)^{w_j}}}{\left(2 - \varphi_j^{(m)}\right)^{w_j} + \varphi^{(m)^{w_j}}}, \frac{2 * \varphi_{ij}^{(r)^{w_j}}}{\left(2 - \varphi_j^{(l)}\right)^{w_j} + \varphi^{(l)^{w_j}}} \right) \tag{7}$$

where

$$\tilde{\varphi}_{ij} = \frac{\ln(\vartheta'_{ij})}{\ln\left(\prod_{i=1}^{m} \vartheta'_{ij}\right)} = \left(\frac{\ln(\vartheta_j'^{(l)})}{\ln\left(\prod_{i=1}^{m} \vartheta_j'^{(r)}\right)}, \frac{\ln(\vartheta_j'^{(m)})}{\ln\left(\prod_{i=1}^{m} \vartheta_j'^{(m)}\right)}, \frac{\ln(\vartheta_j'^{(r)})}{\ln\left(\prod_{i=1}^{m} \vartheta_j'^{(l)}\right)} \right)$$

ξ_{11} , the area's weighted normalized decision matrix's first component, is calculated as follows.

$$\tilde{\varphi}_{11} = \left(\frac{\ln(1.6022)}{\ln(2 * 1.9777 * 2)}, \frac{\ln(1.64)}{\ln(1.64 * 1.6222 * 1.64)}, \frac{\ln(2)}{\ln(1.6022 * 1.4815 * 1.5490)} \right)$$
$$= (0.2279\ 0.3358\ 0.5324)$$

$$\xi_{11} = \left(\frac{2 * 0.2279^{0.2142}}{(2 - 0.5324)^{0.2142} + 0.5324^{0.2142}}, \frac{2 * 0.3358^{0.2142}}{(2 - 0.3358)^{0.2142} + 0.3358^{0.2142}}, \frac{2 * 0.5324^{0.2142}}{(2 - 0.2279)^{0.2142} + 0.2279^{0.2142}} \right)$$

$\xi_{11} = (0{,}7437\ 0.8303\ 0.9400)$

Step 6. Calculation of the final index (Q_i) for the ranking of alternatives.

The Q_i value is calculated by Supplementary Equation (8) and the defuzzification value of $\tilde{Q}_i$ is obtained by Supplementary Equation (6).

$$\tilde{Q}_i = \left(\sum_{j=1}^{n} \tilde{\xi}_{ij} \right) = \left(\sum_{j=1}^{n} \xi_{ij}^{(l)}, \sum_{j=1}^{n} \xi_{ij}^{(m)}, \sum_{j=1}^{n} \xi_{ij}^{(r)} \right) \tag{8}$$

In the case of the areas' ranking,

$$\tilde{Q}_1 = \begin{cases} 0.7437 + 0.7372 + 0.6870 + 0.7012 + 0.6641, \\ 0.8303 + 0.8406 + 0.8471 + 0.8512 + 0.8560, \\ 0.9400 + 0.9656 + 1.0719 + 0.8946 + 1.1356 \end{cases}$$

$\tilde{Q}_1 = (3.5322\ 4.2251\ 5.0077)$ and

$$Q_1 = \frac{3.5322 + 4 * 4.2251 + 5.0077}{6} = 4.2402$$

The alternative with the largest Q_i value is the most important.

After determining the most important criterion based on the weights estimated using the F-LMAW method, the weight sensitivity analysis is performed by changing the weight of the most important criterion (and subsequently the other criteria) to observe the effect of the proposed model on the ranking performance, generating different scenarios where the weights are changed according to a specific parameter (Δx). These are the steps to perform sensitivity analysis based on the weight variation [3],

Step 1: Determination of the weight elasticity coefficient

This variable expresses the relative stability of the criteria weights with respect to specific changes in the weight of the most important criterion during the sensitivity analysis (α_c). Somehow, α_c is the part of the weights of the other criteria apart from the most important one that each criterion has, so when it is multiplied by the Δx parameter to calculate the change in the weight of a criterion (see Supplementary Equation (12)), criteria with a higher value in this coefficient have a greater change in their weight. This value is always defined as 1 for the most important criterion. Supplementary Equation (9) is used for the other criteria. In this way, the weight of the most important criterion will go from 0 in the first scenario to 1 in the last scenario, and the weights of the other criteria will evolve according to this coefficient and the value of the Δx parameter (see next step).

$$\alpha_c = \frac{w_c^0}{1 - w_s^o} \qquad (9)$$

w_c^0: the original value of the changed weight.

w_s^o: the weight of the most important criterion.

Step 2: Determination of the Δx parameter

This parameter represents the amount of change applied to the weight set based on the associated weight elasticity coefficients. . The lower bound for Δx is the original weight of the most important criterion with a negative sign (which will generate a weight of 0 for the most important criterion in the first scenario, according to Supplementary Equation (11), see Supplementary Table S16). The upper bound is calculated so no weight with a negative sign is generated using Supplementary Equation (10).

$$-w_s^o \leq \Delta x \leq w_c^o / \alpha_c \quad (10)$$

Then, to generate different scenarios, Δx is increased by 0.05 (arbitrary value chosen by the authors), except for the second and the last scenarios. For the second scenario, Δx is rounded to the next value multiple of the chosen increase of 0.05, so in one of the scenarios Δx is 0, and the weights coincide with the original ones (in this case, Scenario 6, see Supplementary Table S16). For the last scenario, the value of Δx is the upper bound.

Step 3. Calculation of new criteria weights

Supplementary Equation (11) is used to determine the new weights of the most important criterion, and Supplementary Equation (12) is used to determine the new weights of the other criteria.

$$w_s = w_s^o + \alpha_c \Delta x \quad (11)$$

$$w_c = w_c^o - \alpha_c \Delta x \quad (12)$$

w_s^o: original weight of the criteria subjected to sensitivity analysis, w_c: weight change, w_c^o: the original value of the changed weight.

This new set of criteria weights must always meet $\sum w_s + \sum w_c = 1$.

For example, for Scenario 2 (see Supplementary Table S16), $\Delta x = -0.2000$, the new weights obtained for w_1 and w_2 are calculated using the following parameters,

$w_1^o = 0.2142;\ w_2^o\ = 0.2059;\ \alpha_1 = 1;\ \alpha_2 = 0.2620$

$w_1 = 0.2142 + 1 * (-0.2000) = 0.0142; w_2 = 0.2059 - 0.2620 * (-0.2000) = 0.2583$

For Scenario 11 (see Supplementary Table S16), $\Delta x = 0.2500$, the new weights obtained for w_3 and w_4 are calculated using the following parameters,

$w_3^o = 0.2031;\ w_4^o\ = 0.1865;\ \alpha_3 =\ 0.2585;\ \alpha_4 = 0.2373$

$w_3 = 0.2031 - 0.2585 * 0.2500 = 0.1385; w_4 = 0.1865 - 0.2373 * 0.2500 = 0.1272$

Similar calculations were made for the weights in all scenarios.

References

1. Pamučar, D., Žižović, M., Biswas, S. & Božanić, D. A New Logarithm Methodology of Additive Weights (LMAW) for Multi-Criteria Decision-Making: Application in Logistics. *Facta Universitatis, Series: Mechanical Engineering* **19,** 361–380 (2021).

2. Božanić, D., Pamučar, D., Milić, A., Marinković, D. & Komazec, N. Modification of the Logarithm Methodology of Additive Weights (LMAW) by a Triangular Fuzzy Number and Its Application in Multi-Criteria Decision Making. *Axioms* **11,** 89 (2022).

3. Yazdani, M., Chatterjee, P., Pamucar, D. & Abad, M. D. A risk-based integrated decision-making model for green supplier selection: A case study of a construction company in Spain. *Kybernetes* **49,** 1229–1252 (2020).

Supplementary tables

Supplementary Table S1. Linguistic Evaluations of the Social, Economic and Environmental Areas Made by Experts

	Social	Economic	Environmental
**C1**	VH,VH,VH,VH,H,VH,VH,VH,VH,VH,VH,VH,VH,VH,VH,H,VH	H,H,H,VH,H,H,VH,VH,VH,VH,VH,H,M,VH,VH,H,VH	VH,VH,VH,VH,VH,VH,H,VH,VH,VH,VH,VH,H,H,H,VH,H
**C2**	H,H,VH,VH,VH,H,VH,H,VH,VH,H,VH,VH,VH,VH,H,VH	H,H,H,VH,M,M,VH,H,VH,VH,VH,M,M,VH,H,M,VH	VH,H,VH,VH,VH,VH,H,VH,VH,VH,VH,VH,VH,H,H,H,VH
**C3**	M,H,H,VH,VH,VH,H,H,VH,M,VS,S,M,H,H,M,M	M,H,H,H,VH,H,VH,H,H,VS,M,M,H,H,M,H,H	M,VS,M,M,M,M,H,S,M,VS,M,S,M,H,M,M,M
**C4**	H,M,H,H,H,H,H,M,H,M,M,S,M,M,H,S,S	H,H,H,H,H,M,H,M,VH,M,M,M,H,M,H,S,S	H,H,H,M,H,H,M,S,M,VS,H,S,H,M,H,M,S
**C5**	S,S,VS,M,H,M,S,H,H,VH,S,M,M,M,M,H,H	S,S,VS,S,H,M,M,S,H,VH,H,H,M,M,H,VH,M	S,S,M,S,M,S,VS,VS,M,H,H,M,M,M,M,M,S

Supplementary Table S2. Linguistic Evaluation of SDGs Made by Experts

SDGs	C1	C2	C3	C4	C5
1	VH,H,VH,VH,H,H,VH,VH,VH,H,VH,VH,M,VH,VH,H,VH	H,H,VH,H,M,H,VH,VH,H,H,H,VH,M,VH,VH,H,H	S,VH,H,VH,VH,VH,VH,H,VH,M,S,M,S,VH,VH,M,M	M,VH,H,VH,H,M,H,S,VH,S,M,M,M,H,M,S,S	S,M,VS,VH,VH,M,M,H,M,H,H,M,H,H,H,VH,M
2	H,H,VH,H,VH,H,VH,VH,H,H,H,VH,M,VH,H,S,H	H,H,H,M,H,VH,VH,M,VH,H,H,VH,M,VH,H,S,M	M,H,VH,H,H,H,VH,VH,VH,S,M,M,M,VH,VH,M,M	M,H,H,H,H,M,H,S,VH,S,M,M,M,H,M,S,S	S,H,S,VH,H,H,S,H,H,M,H,M,H,H,VH,H,H
3	VH,VH,VH,VH,VH,VH,VH,VH,VH,H,VH,VH,VH,VH,VH,M,H	VH,VH,VH,VH,VH,VH,VH,VH,VH,H,VH,VH,VH,VH,VH,M,H	M,M,VH,VH,H,M,H,M,M,M,H,S,S,H,H,M,M	M,H,H,VH,H,H,H,S,M,S,H,S,VS,M,M,VS,S	M,S,VS,S,H,S,S,H,S,M,VH,H,M,M,H,M,M
4	H,VH,VH,VH,VH,VH,H,VH,VH,VH,VH,VH,H,VH,VH,VH,VH	H,H,VH,VH,H,VH,H,VH,H,VH,VH,VH,VH,VH,VH,H,VH	S,H,VH,VH,VH,H,H,S,M,S,H,VS,S,VH,VH,H,M	S,VH,M,VH,H,M,H,VS,M,M,H,S,VS,M,H,S,S	M,VH,M,S,H,S,M,M,H,M,VH,H,H,M,M,H,H
5	VH,H,VH,VH,H,VH,M,VH,VH,VH,VH,VH,H,VH,H,H,M	VH,H,VH,H,M,H,H,H,VH,VH,H,VH,H,VH,VH,H,S	H,M,VH,VH,VH,M,M,H,VS,H,S,S,M,H,H,M,S	H,VH,H,M,H,H,H,VS,VS,H,M,M,M,M,M,M,S	M,H,M,M,VH,H,VS,H,S,H,M,H,M,M,H,M,H
10	H,H,VH,VH,VH,VH,H,VH,VH,VH,VH,VH,VH,VH,VH,VH,VH	M,VH,VH,VH,VH,VH,VH,VH,VH,VH,VH,VH,VH,VH,VH,VH,VH	M,M,M,H,M,M,H,VS,S,M,M,VS,S,H,VH,H,M	S,VH,H,M,M,M,H,VS,S,H,M,VS,S,M,H,S,S	M,S,VS,VS,M,S,S,M,S,H,M,H,M,M,M,H,M
7	H,H,VH,VH,VH,VH,M,H,H,M,VH,VH,VH,H,VH,M,M	M,M,VH,H,H,VH,VH,VH,H,M,VH,VH,VH,VH,VH,M,M	H,M,M,H,VH,M,M,S,M,S,M,S,M,M,H,H,M	M,H,M,H,H,M,M,S,M,S,S,VS,H,M,S,VS,S	H,H,M,S,VH,VS,S,S,VS,S,H,S,S,M,S,M,H
8	VH,VH,VH,H,VH,H,VH,VH,VH,VH,VH,VH,H,VH,VH,H,VH	H,H,VH,VH,H,H,VH,VH,VH,VH,VH,VH,VH,H,H,H,VH	M,H,H,H,VH,VH,VH,S,M,S,M,M,M,H,M,H,H	S,H,H,H,H,H,H,M,H,M,M,H,M,M,S,S	M,M,M,M,VH,H,M,H,M,M,H,VH,H,H,M,VH,H
9	H,H,H,VH,H,H,H,VH,H,M,VH,M,H,H,VH,M,M	VH,M,H,H,H,M,H,VH,M,M,H,S,H,H,VH,M,M	M,S,VH,H,M,H,H,S,S,H,H,VH,S,H,H,H,M	H,M,H,H,M,M,M,S,S,H,H,M,M,M,H,VS,S	H,VS,S,M,M,S,VS,S,S,H,H,VH,S,M,H,H,M
11	VH,H,VH,VH,H,VH,H,VH,VH,H,VH,VH,H,H,VH,H,VH	H,M,H,H,H,VH,VH,H,VH,H,VH,VH,H,H,H,H,VH	M,S,M,H,H,S,M,S,S,S,H,S,S,H,S,M,H	H,M,M,M,M,M,M,VS,S,M,M,S,S,M,S,S,S	M,VS,M,M,H,VS,VS,S,M,M,M,M,M,S,M,H,S
12	VH,H,VH,VH,H,VH,VH,VH,VH,H,VH,VH,M,VH,H,VH,S	H,VH,H,H,VH,VH,VH,VH,VH,H,VH,VH,M,VH,M,H,S	H,VS,M,H,H,M,H,VS,VS,M,M,S,S,VH,M,S,S	M,M,M,VH,M,M,M,VS,VS,M,M,S,M,M,M,VS,S	M,VS,M,VS,M,S,S,VS,VS,M,M,M,M,M,M,H,H
6	VH,VH,VH,VH,VH,VH,H,VH,VH,S,VH,VH,VH,VH,VH,H,VH	H,VH,VH,VH,VH,VH,VH,VH,VH,S,VH,VH,VH,VH,VH,H,VH	M,M,H,VH,VH,M,M,VH,S,VH,M,H,M,H,VH,M,M	H,VH,H,VH,VH,M,M,S,S,VH,M,H,M,M,M,VS,S	M,VS,H,VS,VH,S,S,S,S,M,H,H,M,H,VH,M,S
13	VH,H,VH,VH,VH,VH,H,VH,VH,VH,VH,VH,H,VH,H,H,H	VH,H,VH,VH,VH,VH,VH,H,VH,VH,VH,VH,VH,VH,H,H,VH	H,M,H,S,VH,H,M,VH,M,S,H,S,M,M,M,H,M	H,VH,H,S,VH,H,H,S,M,S,H,M,H,M,M,M,M	M,S,M,VS,VH,S,S,S,VS,M,H,M,M,M,H,M,S
14	VH,M,VH,H,VH,VH,H,H,VH,H,VH,VH,VH,H,H,S,M	VH,M,VH,H,H,VH,VH,H,VH,H,VH,VH,VH,M,M,S,M	M,VS,VH,M,M,S,H,VS,VS,S,H,S,M,M,M,S,S	H,M,H,S,VH,H,H,VS,VS,S,H,M,M,M,M,VS,S	S,VS,H,VS,M,VSS,VS,VS,H,H,M,M,M,S,M,H
15	VH,H,VH,VH,VH,VH,VH,VH,VH,VH,M,VH,VH,H,H,VH,M,M	VH,M,VH,VH,H,VH,VH,VH,VH,H,M,VH,VH,H,M,H,M,H	M,H,H,H,H,M,M,H,M,H,M,M,S,M,M,M,S,S	M,H,H,M,VH,H,M,H,M,H,S,M,M,M,M,M,S,S	S,S,H,VS,H,S,S,S,S,S,H,H,M,M,M,M,M,H
16	VH,VH,VH,VH,VH,VH,VH,VH,VH,VH,VH,VH,VH,VH,VH,VH,VH	VH,H,VH,VH,VH,VH,VH,VH,VH,VH,VH,VH,VH,VH,H,VH	S,M,M,H,VH,H,VH,H,M,H,M,M,M,H,M,M,H	S,M,M,H,H,H,H,H,S,H,M,M,H,H,M,S,M	M,S,H,VS,VH,H,M,H,M,M,H,H,M,M,H,VH,VH
17	H,H,VH,H,VH,VH,VH,VH,VH,H,VH,VH,H,H,VH,M,H	VH,H,VH,H,VH,VH,VH,VH,VH,H,VH,VH,H,H,VH,M,M	M,M,H,M,VH,H,H,M,M,S,M,M,M,H,H,S,H	M,S,H,M,H,H,H,M,M,M,M,M,H,M,H,S,M	S,S,H,S,H,H,S,M,M,H,H,H,VH,M,H,S,S

Supplementary Table S3. Aggregated Decision Matrix of the Areas

	**C1**			**C2**			**C3**			**C4**			**C5**		
	l	m	r	l	m	r	l	m	r	l	m	r	l	m	r
**Soc**	7.4449	7.9118	12.3621	6.5699	7.9118	12.3621	3.6103	5.7647	9.6434	2.5882	5.3529	9.1066	2.1985	4.4320	7.8952
**Eco**	5.9522	7.6912	12.0864	4.7794	6.7868	10.9467	3.7739	6.3621	10.4099	2.9982	5.7371	9.6048	2.4301	4.5882	8.1048
**Env**	6.7868	7.9118	12.3621	6.7721	7.9118	12.3621	1.7426	3.7629	6.9926	2.4449	4.9596	8.5938	1.4099	3.2647	6.3107
**max**			12.3621			12.3621			10.4099			9.6048			8.1048

Supplementary Table S4. Aggregated Decision Matrix of the SDGs

**SDGs**	**C1**			**C2**			**C3**			**C4**			**C5**		
	l	m	r	l	m	l	m	r	l	m	l	m	r	l	m
**1**	3.5851	3.9407	4.9408	3.2313	3.8815	4.8817	2.8672	3.4063	4.4075	2.3389	3.1704	4.1719	2.5782	3.3464	4.3479
**2**	3.1704	3.8214	4.8218	2.9331	3.6441	4.6447	2.8723	3.5262	4.5269	2.2245	3.1704	4.1719	2.6374	3.5241	4.5253
**3**	3.7622	3.9407	4.9408	3.7622	3.9407	4.9408	2.4025	3.2899	4.2909	2.1039	2.9293	3.9323	1.9871	2.8736	3.8758
**4**	3.8223	4.0000	5.0000	3.6451	4.0000	5.0000	2.5710	3.2256	4.2279	2.1022	2.8710	3.8739	2.4615	3.3486	4.3496
**5**	3.4662	3.8815	4.8817	3.2290	3.8214	4.8218	2.3979	3.1681	4.1701	2.2849	3.1083	4.1106	2.4040	3.2877	4.2892
**10**	3.8223	4.0000	5.0000	3.8806	3.9407	4.9408	2.1074	2.9318	3.9342	1.9270	2.7533	3.7564	1.8150	2.6967	3.6992
**7**	3.2290	3.7632	4.7635	3.2279	3.7041	4.7045	2.1676	3.1131	4.1142	1.8129	2.6953	3.6982	1.8068	2.6346	3.6380
**8**	3.7632	4.0000	5.0000	3.5861	4.0000	5.0000	2.5782	3.4074	4.4084	2.2262	3.2290	4.2305	2.6994	3.5272	4.5277
**9**	2.9951	3.7632	4.7635	2.6980	3.5262	4.5269	2.4585	3.3464	4.3479	2.1092	3.0510	4.0530	1.9852	2.8115	3.8146
**11**	3.5861	4.0000	5.0000	3.2910	3.9407	4.9408	1.8109	2.8154	3.8175	1.6405	2.5825	3.5841	1.7573	2.5782	3.5810
**12**	3.4641	3.8214	4.8218	3.2866	3.7622	4.7628	1.9270	2.6926	3.6962	1.8150	2.5796	3.5820	1.7573	2.5176	3.5209
**6**	3.7012	3.8806	4.8809	3.7012	3.8806	4.8809	2.6953	3.4085	4.4092	2.3964	3.1095	4.1115	2.0418	2.8115	3.8146
**13**	3.6451	4.0000	5.0000	3.7632	4.0000	5.0000	2.3421	3.2301	4.2314	2.4010	3.2888	4.2900	1.8109	2.6374	3.6401
**14**	3.2279	3.7622	4.7628	3.1083	3.6441	4.6447	1.7511	2.5176	3.5209	2.0454	2.8102	3.8137	1.7531	2.4540	3.4588
**15**	3.4652	3.8223	4.8226	3.2290	3.7632	4.7635	2.1693	3.1716	4.1727	2.2262	3.1716	4.1727	1.8708	2.8141	3.8166
**16**	4.0000	4.0000	5.0000	3.8815	4.0000	5.0000	2.5220	3.4085	4.4092	2.2865	3.2888	4.2900	2.5782	3.3464	4.3479
**17**	3.4673	3.9407	4.9408	3.4662	3.8815	4.8817	2.3452	3.2899	4.2909	2.2295	3.2313	4.2322	2.1625	3.1095	4.1115
**max**			5.0000			5.0000			4.5269			4.2900			4.5277

Supplementary Table S5. Normalized Decision Matrix of the Areas

	**C1**			**C2**			**C3**			**C4**			**C5**		
	l	m	r	l	m	r	l	m	r	l	m	r	l	m	r
**Soc**	1.6022	1.6400	2.0000	1.5315	1.6400	2.0000	1.2920	1.4663	1.7801	1.2094	1.4330	1.7367	1.1778	1.3585	1.6387
**Eco**	1.4815	1.6222	1.9777	1.3866	1.5490	1.8855	1.3053	1.5146	1.8421	1.2425	1.4641	1.7770	1.1966	1.3712	1.6556
**Env**	1.5490	1.6400	2.0000	1.5478	1.6400	2.0000	1.1410	1.3044	1.5657	1.1978	1.4012	1.6952	1.1141	1.2641	1.5105

Supplementary Table S6. Normalized Decision Matrix of the SDGs

**SDGs**		**C1**			**C2**			**C3**			**C4**			**C5**	
	l	m	r	l	m	l	m	r	l	m	l	m	r	l	m
**1**	0.0465	0.0593	0.0756	0.0433	0.0593	0.0772	0.0440	0.0623	0.0967	0.0384	0.0615	0.1008	0.0420	0.0650	0.1043
**2**	0.0423	0.0579	0.0743	0.0401	0.0565	0.0745	0.0440	0.0640	0.0986	0.0368	0.0615	0.1008	0.0428	0.0676	0.1074
**3**	0.0483	0.0593	0.0756	0.0487	0.0600	0.0779	0.0380	0.0606	0.0948	0.0351	0.0577	0.0964	0.0338	0.0576	0.0957
**4**	0.0489	0.0599	0.0762	0.0476	0.0607	0.0786	0.0402	0.0597	0.0937	0.0351	0.0568	0.0953	0.0404	0.0650	0.1043
**5**	0.0453	0.0586	0.0749	0.0433	0.0586	0.0765	0.0380	0.0588	0.0928	0.0377	0.0605	0.0997	0.0396	0.0641	0.1033
**10**	0.0489	0.0599	0.0762	0.0499	0.0600	0.0779	0.0341	0.0553	0.0888	0.0326	0.0549	0.0931	0.0313	0.0547	0.0923
**7**	0.0429	0.0572	0.0736	0.0433	0.0572	0.0752	0.0349	0.0580	0.0918	0.0310	0.0540	0.0920	0.0311	0.0537	0.0911
**8**	0.0483	0.0599	0.0762	0.0470	0.0607	0.0786	0.0403	0.0623	0.0967	0.0368	0.0624	0.1018	0.0436	0.0677	0.1075
**9**	0.0404	0.0572	0.0736	0.0375	0.0551	0.0731	0.0388	0.0614	0.0957	0.0352	0.0596	0.0986	0.0337	0.0566	0.0945
**11**	0.0465	0.0599	0.0762	0.0439	0.0600	0.0779	0.0300	0.0535	0.0868	0.0284	0.0521	0.0898	0.0304	0.0527	0.0900
**12**	0.0453	0.0579	0.0743	0.0439	0.0579	0.0759	0.0316	0.0516	0.0847	0.0310	0.0521	0.0897	0.0304	0.0517	0.0889
**6**	0.0477	0.0586	0.0749	0.0481	0.0593	0.0772	0.0418	0.0623	0.0967	0.0392	0.0605	0.0997	0.0346	0.0566	0.0945
**13**	0.0471	0.0599	0.0762	0.0487	0.0607	0.0786	0.0373	0.0597	0.0938	0.0392	0.0633	0.1029	0.0312	0.0537	0.0912
**14**	0.0429	0.0572	0.0736	0.0420	0.0565	0.0745	0.0291	0.0489	0.0815	0.0343	0.0558	0.0942	0.0303	0.0506	0.0876
**15**	0.0453	0.0579	0.0743	0.0433	0.0579	0.0759	0.0349	0.0589	0.0928	0.0368	0.0615	0.1008	0.0321	0.0566	0.0946
**16**	0.0506	0.0599	0.0762	0.0499	0.0607	0.0786	0.0396	0.0623	0.0967	0.0377	0.0633	0.1029	0.0420	0.0650	0.1043
**17**	0.0453	0.0593	0.0756	0.0457	0.0593	0.0772	0.0373	0.0606	0.0948	0.0369	0.0624	0.1019	0.0363	0.0613	0.1000

Supplementary Table S7. Linguistic Evaluation of Criteria by Experts

	**C1**	**C2**	**C3**	**C4**	**C5**
**e1**	AH	VH	E	H	VH
**e2**	AH	H	VH	MH	MH
**e3**	AH	H	VH	H	AH
**e4**	AH	AH	H	E	VH
**e5**	H	H	VH	MH	VH
**e6**	AH	H	VH	MH	H
**e7**	AH	VH	VH	H	VH
**e8**	AH	AH	VH	MH	H
**e9**	AH	VH	H	MH	AH
**e10**	AH	AH	AH	MH	MH
**e11**	AH	M	M	MH	E
**e12**	H	VH	H	MH	H
**e13**	H	VH	MH	E	MH
**e14**	H	AH	H	MH	E
**e15**	H	MH	VH	H	VH
**e16**	H	MH	VH	M	VL
**e17**	AH	AH	VH	E	L

Supplementary Table S8. Numerical Values according to the Linguistic Evaluation of Criteria by Experts

Experts	C1			C2			C3			C4			C5		
	l	m	r	l	m	r	l	m	r	l	m	r	l	m	r
**e1**	4.5	5	5	4	4.5	5	2.5	3	3.5	3.5	4	4.5	4	4.5	5
**e2**	4.5	5	5	3.5	4	4.5	4	4.5	5	3	3.5	4	3	3.5	4
**e3**	4.5	5	5	3.5	4	4.5	4	4.5	5	3.5	4	4.5	4.5	5	5
**e4**	4.5	5	5	4.5	5	5	3.5	4	4.5	2.5	3	3.5	4	4.5	5
**e5**	3.5	4	4.5	3.5	4	4.5	4	4.5	5	3	3.5	4	4	4.5	5
**e6**	4.5	5	5	3.5	4	4.5	4	4.5	5	3	3.5	4	3.5	4	4.5
**e7**	4.5	5	5	4	4.5	5	4	4.5	5	3.5	4	4.5	4	4.5	5
**e8**	4.5	5	5	4.5	5	5	4	4.5	5	3	3.5	4	3.5	4	4.5
**e9**	4.5	5	5	4	4.5	5	3.5	4	4.5	3	3.5	4	4.5	5	5
**e10**	4.5	5	5	4.5	5	5	4.5	5	5	3	3.5	4	3	3.5	4
**e11**	4.5	5	5	2	2.5	3	2	2.5	3	3	3.5	4	2.5	3	3.5
**e12**	3.5	4	4.5	4	4.5	5	3.5	4	4.5	3	3.5	4	3.5	4	4.5
**e13**	3.5	4	4.5	4	4.5	5	3	3.5	4	2.5	3	3.5	3	3.5	4
**e14**	3.5	4	4.5	4.5	5	5	3.5	4	4.5	3	3.5	4	2.5	3	3.5
**e15**	3.5	4	4.5	3	3.5	4	4	4.5	5	3.5	4	4.5	4	4.5	5
**e16**	3.5	4	4.5	3	3.5	4	4	4.5	5	2	2.5	3	1	1.5	2
**e17**	4.5	5	5	4.5	5	5	4	4.5	5	2.5	3	3.5	1.5	2	2.5

Supplementary Table S9. Fuzzy Relationship Vectors

Experts	C1			C2			C3			C4			C5		
	l	m	r	l	m	r	l	m	r	l	m	r	l	m	r
**e1**	9	10	10	8	9	10	5	6	7	7	8	9	8	9	10
**e2**	9	10	10	7	8	9	8	9	10	6	7	8	6	7	8
**e3**	9	10	10	7	8	9	8	9	10	7	8	9	9	10	10
**e4**	9	10	10	9	10	10	7	8	9	5	6	7	8	9	10
**e5**	7	8	9	7	8	9	8	9	10	6	7	8	8	9	10
**e6**	9	10	10	7	8	9	8	9	10	6	7	8	7	8	9
**e7**	9	10	10	8	9	10	8	9	10	7	8	9	8	9	10
**e8**	9	10	10	9	10	10	8	9	10	6	7	8	7	8	9
**e9**	9	10	10	8	9	10	7	8	9	6	7	8	9	10	10
**e10**	9	10	10	9	10	10	9	10	10	6	7	8	6	7	8
**e11**	9	10	10	4	5	6	4	5	6	6	7	8	5	6	7
**e12**	7	8	9	8	9	10	7	8	9	6	7	8	7	8	9
**e13**	7	8	9	8	9	10	6	7	8	5	6	7	6	7	8
**e14**	7	8	9	9	10	10	7	8	9	6	7	8	5	6	7
**e15**	7	8	9	6	7	8	8	9	10	7	8	9	8	9	10
**e16**	7	8	9	6	7	8	8	9	10	4	5	6	2	3	4
**e17**	9	10	10	9	10	10	8	9	10	5	6	7	3	4	5

Supplementary Table S10. Weight Coefficient Vectors of Criteria

**Experts**		**C1**			**C2**			**C3**			**C4**			**C5**	
	l	m	r	l	m	r	l	m	r	l	m	r	l	m	r
**e1**	0.19883	0.21788	0.23232	0.18817	0.20791	0.23232	0.14564	0.16954	0.19633	0.17609	0.19676	0.22169	0.18817	0.20791	0.23232
**e2**	0.20045	0.21990	0.23481	0.17753	0.19859	0.22407	0.18971	0.20984	0.23481	0.16346	0.18584	0.21206	0.16346	0.18584	0.21206
**e3**	0.19441	0.21007	0.22213	0.17217	0.18971	0.21197	0.18399	0.20045	0.22213	0.17217	0.18971	0.21197	0.19441	0.21007	0.22213
**e4**	0.19883	0.21573	0.22959	0.19883	0.21573	0.22959	0.17609	0.19482	0.21908	0.14564	0.16787	0.19402	0.18817	0.20586	0.22959
**e5**	0.17564	0.19806	0.22324	0.17564	0.19806	0.22324	0.18769	0.20927	0.23394	0.16172	0.18534	0.21127	0.18769	0.20927	0.23394
**e6**	0.19832	0.21713	0.23118	0.17564	0.19609	0.22060	0.18769	0.20720	0.23118	0.16172	0.18350	0.20877	0.17564	0.19609	0.22060
**e7**	0.19261	0.20983	0.22180	0.18229	0.20023	0.22180	0.18229	0.20023	0.22180	0.17058	0.18949	0.21165	0.18229	0.20023	0.22180
**e8**	0.19645	0.21266	0.22549	0.19645	0.21266	0.22549	0.18592	0.20293	0.22549	0.16020	0.17972	0.20364	0.17398	0.19205	0.21517
**e9**	0.19645	0.21266	0.22549	0.18592	0.20293	0.22549	0.17398	0.19205	0.21517	0.16020	0.17972	0.20364	0.19645	0.21266	0.22549
**e10**	0.19854	0.21321	0.22629	0.19854	0.21321	0.22629	0.19854	0.21321	0.22629	0.16191	0.18018	0.20436	0.16191	0.18018	0.20436
**e11**	0.22169	0.24868	0.27507	0.13987	0.17382	0.21404	0.13987	0.17382	0.21404	0.18078	0.21016	0.24841	0.16238	0.19351	0.23246
**e12**	0.17732	0.20030	0.22631	0.18949	0.21165	0.23716	0.17732	0.20030	0.22631	0.16328	0.18744	0.21418	0.17732	0.20030	0.22631
**e13**	0.18350	0.20877	0.23835	0.19609	0.22060	0.24978	0.16896	0.19537	0.22558	0.15177	0.17989	0.21109	0.16896	0.19537	0.22558
**e14**	0.18148	0.20388	0.23152	0.20492	0.22576	0.24263	0.18148	0.20388	0.23152	0.16710	0.19079	0.21911	0.15010	0.17568	0.20504
**e15**	0.17564	0.19806	0.22324	0.16172	0.18534	0.21127	0.18769	0.20927	0.23394	0.17564	0.19806	0.22324	0.18769	0.20927	0.23394
**e16**	0.19943	0.23284	0.27825	0.18363	0.21789	0.26334	0.21312	0.24603	0.29159	0.14208	0.18022	0.22690	0.07104	0.12302	0.17556
**e17**	0.21000	0.23071	0.25077	0.21000	0.23071	0.25077	0.19874	0.22015	0.25077	0.15382	0.17953	0.21193	0.10500	0.13890	0.17528

Supplementary Table S11. Aggregated Fuzzy Vector of Weight Coefficients.

**C1**	**C2**	**C3**	**C4**	**C5**	**C1**	**C2**	**C3**	**C4**	**C5**	**C1**	**C2**	**C3**	**C4**	**C5**
l	m	r	l	m	l	m	r	l	m	l	m	r	l	m
0.19407	0.21470	0.23501	0.18448	0.20590	0.22996	0.18105	0.20280	0.22936	0.16281	0.18612	0.21398	0.16655	0.19027	0.21711

Supplementary Table S12. Weighted Normalized Matrix of the Areas

	C1			C2			C3			C4			C5		
	l	m	r	l	m	r	l	m	r	l	m	r	l	m	r
**Soc**	0.7437	0.8303	0.9400	0.7372	0.8406	0.9656	0.6870	0.8471	1.0719	0.7012	0.8512	0.8946	0.6641	0.8560	1.1356
**Eco**	0.7159	0.8275	0.9483	0.7011	0.8259	0.9649	0.6919	0.8571	1.0817	0.7171	0.8576	0.8939	0.6761	0.8594	1.1334
**Env**	0.7319	0.8303	0.9447	0.7410	0.8406	0.9641	0.6046	0.8048	1.0636	0.6963	0.8442	0.8900	0.6135	0.8265	1.1262

Supplementary Table S13. Weighted Normalized Matrix of the SDGs

**SDGs**		**C1**			**C2**			**C3**			**C4**			**C5**	
	l	m	r	l	m	l	m	r	l	m	l	m	r	l	m
**1**	0.6008	0.6429	0.6878	0.6042	0.6556	0.7060	0.6017	0.6643	0.7424	0.6122	0.6889	0.7768	0.6145	0.6877	0.7727
**2**	0.5893	0.6406	0.6893	0.5961	0.6511	0.7040	0.6011	0.6668	0.7453	0.6075	0.6889	0.7787	0.6155	0.6913	0.7762
**3**	0.6056	0.6429	0.6861	0.6187	0.6567	0.7021	0.5851	0.6618	0.7458	0.6038	0.6835	0.7743	0.5928	0.6771	0.7698
**4**	0.6069	0.6440	0.6869	0.6153	0.6577	0.7044	0.5922	0.6604	0.7417	0.6042	0.6821	0.7727	0.6101	0.6878	0.7745
**5**	0.5978	0.6418	0.6876	0.6045	0.6545	0.7047	0.5857	0.6590	0.7427	0.6104	0.6876	0.7761	0.6082	0.6865	0.7738
**10**	0.6069	0.6440	0.6869	0.6218	0.6567	0.7011	0.5747	0.6534	0.7409	0.5968	0.6792	0.7726	0.5854	0.6726	0.7680
**7**	0.5915	0.6395	0.6874	0.6052	0.6522	0.7021	0.5762	0.6578	0.7449	0.5915	0.6777	0.7732	0.5855	0.6709	0.7663
**8**	0.6053	0.6440	0.6874	0.6137	0.6577	0.7049	0.5913	0.6644	0.7463	0.6071	0.6902	0.7802	0.6177	0.6914	0.7754
**9**	0.5840	0.6395	0.6899	0.5886	0.6487	0.7042	0.5870	0.6630	0.7465	0.6032	0.6863	0.7775	0.5932	0.6755	0.7680
**11**	0.6005	0.6440	0.6890	0.6057	0.6567	0.7066	0.5606	0.6504	0.7431	0.5829	0.6747	0.7735	0.5833	0.6694	0.7656
**12**	0.5981	0.6406	0.6863	0.6066	0.6534	0.7028	0.5676	0.6471	0.7370	0.5925	0.6746	0.7696	0.5837	0.6677	0.7637
**6**	0.6044	0.6418	0.6854	0.6175	0.6556	0.7014	0.5956	0.6644	0.7447	0.6149	0.6876	0.7743	0.5959	0.6755	0.7670
**13**	0.6022	0.6440	0.6885	0.6184	0.6577	0.7033	0.5830	0.6605	0.7452	0.6139	0.6914	0.7789	0.5857	0.6710	0.7663
**14**	0.5914	0.6395	0.6874	0.6018	0.6511	0.7020	0.5596	0.6421	0.7350	0.6020	0.6806	0.7720	0.5840	0.6659	0.7617
**15**	0.5981	0.6406	0.6863	0.6048	0.6534	0.7034	0.5759	0.6591	0.7465	0.6075	0.6890	0.7787	0.5875	0.6756	0.7703
**16**	0.6114	0.6440	0.6854	0.6214	0.6577	0.7023	0.5891	0.6644	0.7471	0.6093	0.6914	0.7807	0.6145	0.6877	0.7727
**17**	0.5975	0.6429	0.6889	0.6111	0.6556	0.7036	0.5827	0.6618	0.7467	0.6073	0.6902	0.7802	0.5993	0.6826	0.7731

Supplementary Table S14. Final Index for the Ranking of the Areas (Fuzzy and Defuzzified)

	Qi			Qi
	l	m	u	
Soc	3.5332	4.2251	5.0077	4.2402
Eco	3.5021	4.2274	5.0224	4.2390
Env	3.3873	4.1463	4.9887	4.1602

Supplementary Table S15. Weight Elasticity Coefficient for Changing Weights

Criteria	Calculated Weights	α_c	Δx
C1	0.2142	1	
C2	0.2059	0.2059/(1-0.2142) =0.2620	0.2059/0.2620=0.7858
C3	0.2031	0.2031/(1-0.2142)=0.2585	0.2031/0.2585=0.7858
C4	0.1865	0.1865/(1-0.2142)=0.2373	0.1865/0.2373=0.7858
C5	0.1904	0.1904/(1-0.2142)=0.2423	0.1904/0.2423=0.7858

Supplementary Table S16. New Criteria Weights

Scenario	Δx	w1	w2	w3	w4	w5	Total
Sc1	-0.2142	0.0000	0.2620	0.2585	0.2373	0.2423	1
Sc2	-0.2000	0.0142	0.2583	0.2548	0.2340	0.2389	1
Sc3	-0.1500	0.0642	0.2452	0.2419	0.2221	0.2267	1
Sc4	-0.1000	0.1142	0.2321	0.2289	0.2102	0.2146	1
Sc5	-0.0500	0.1642	0.2190	0.2160	0.1984	0.2025	1
Sc6	0.0000	0.2142	0.2059	0.2031	0.1865	0.1904	1
Sc7	0.0500	0.2642	0.1928	0.1902	0.1746	0.1783	1
Sc8	0.1000	0.3142	0.1797	0.1773	0.1628	0.1662	1
Sc9	0.1500	0.3642	0.1666	0.1643	0.1509	0.1541	1
Sc10	0.2000	0.4142	0.1535	0.1514	0.1390	0.1419	1
Sc11	0.2500	0.4642	0.1404	0.1385	0.1272	0.1298	1
Sc12	0.3000	0.5142	0.1273	0.1256	0.1153	0.1177	1
Sc13	0.3500	0.5642	0.1142	0.1126	0.1034	0.1056	1
Sc14	0.4000	0.6142	0.1011	0.0997	0.0916	0.0935	1
Sc15	0.4500	0.6642	0.0880	0.0868	0.0797	0.0814	1
Sc16	0.5000	0.7142	0.0749	0.0739	0.0678	0.0692	1
Sc17	0.5500	0.7642	0.0618	0.0609	0.0560	0.0571	1
Sc18	0.6000	0.8142	0.0487	0.0480	0.0441	0.0450	1
Sc19	0.6500	0.8642	0.0356	0.0351	0.0322	0.0329	1
Sc20	0.7000	0.9142	0.0225	0.0222	0.0204	0.0208	1
Sc21	0.7500	0.9642	0.0094	0.0093	0.0085	0.0087	1
Sc22	0.7858	1.0000	0.0000	0.0000	0.0000	0.0000	1

Supplementary Table S17. SRC Values of the Tested Methods

	F-LMAW	F-SAW	F-TOPSIS	F-WASPAS	F-ARAS
F-LMAW	1.000	0.985	0.995	0.990	0.990
F-SAW		1.000	0.980	0.995	0.995
F-TOPSIS			1.000	0.985	0.985
F-WASPAS				1.000	1.000
F-ARAS					1.000
